# Electrophilic activation of water by a carbene catalyzed by a copper surface

**DOI:** 10.1039/d6sc00488a

**Published:** 2026-06-06

**Authors:** Yunjun Cao, Joel Mieres-Perez, Julien Frederic Rowen, Elsa Sanchez-Garcia, Wolfram Sander, Karina Morgenstern

**Affiliations:** a Physical Chemistry I, Ruhr-Universität Bochum D-44801 Bochum Germany karina.morgenstern@rub.de; b Computational Bioengineering, Technische Universität Dortmund D-44227 Dortmund Germany elsa.sanchez@tu-dortmund.de; c Organic Chemistry II, Ruhr-Universität Bochum D-44801 Bochum Germany wolfram.sander@rub.de

## Abstract

Carbenes are among the most versatile organic intermediates, capable of exhibiting radical, electrophilic, or nucleophilic reactivity. Harnessing this versatility for synthesis requires precise control over carbene philicity. In reactions with protic molecules such as water or alcohols, free carbenes are generally nucleophilic and react exclusively *via* proton transfer to form carbenium ions. Here we demonstrate a fundamentally different reactivity mode: electrophilic activation of water by an archetypal aryl carbene catalyzed by a copper surface. Surface infrared spectroscopy, bond-resolved scanning tunneling microscopy, and theoretical modeling reveal that the carbene reacts with the water as an electrophile, forming a surface-stabilized ylide that is subsequently deprotonated. This heterogeneous pathway contrasts sharply with the behavior of metal-free carbenes in solution and cryogenic matrices, where reaction proceeds exclusively through proton transfer from the water to the carbene. These results provide molecular-level insight into surface-catalyzed modulation of carbene reactivity and establish heterogeneous catalysis as a platform for accessing otherwise inaccessible carbene–water reaction pathways.

## Abstract 2

Adsorption on a Cu(111) surface alters the philicity of a carbene to electrophilic. Its reaction with water is followed by bond-resolved scanning tunneling microscopy, surface infrared spectroscopy, and theoretical modeling.

## Graphical abstract

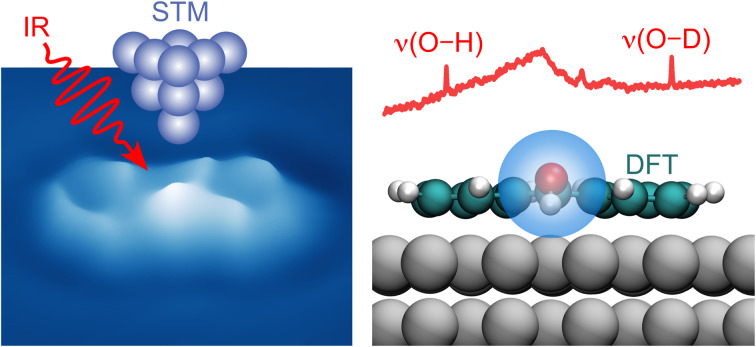

## Introduction

Carbenes are among the most versatile reactive intermediates in organic chemistry. Depending on their substitution pattern and local environment, carbenes may adopt singlet or triplet spin ground states, display electrophilic, nucleophilic, or radical reactivity, and exhibit lifetimes ranging from fleeting to persistent under laboratory conditions.^1^ This versatility renders carbenes key intermediates in organic synthesis, functioning as reactive electrophiles,^2^ strong neutral bases and nucleophiles (most notably N-heterocyclic carbenes, NHCs),^3^ or ligands in a wide range of metal complexes.^4^

A key descriptor for understanding the reactivity of carbenes in their singlet states is their philicity (electrophilicity and nucleophilicity). NHCs exhibit a high nucleophilicity and basicity, but only low to moderate electrophilicity.^5^ This limits the reactivity of these stable and often isolable carbenes. In contrast, the singlet state of diphenylcarbene S-1 shows both very high nucleo- and electrophilicity, resulting in extreme reactivity. In protic solvents such as methanol, S-1 is protonated on an ultrafast time scale^6^ and is markedly more basic and nucleophilic than NHCs.^7^

A computational study of carbene–coinage-metal surface interactions reveals fundamental differences between electrophilic and nucleophilic carbenes.^8^ Imidazolylidene, an archetypal N-heterocyclic carbene (NHC), binds primarily *via* its high-lying, doubly occupied σ lone pair at the carbene center (HOMO) and therefore adsorbs approximately upright on metal surfaces. Its π-type LUMO lies relatively high in energy, leading to low electrophilicity and generally modest chemical reactivity for NHCs. In contrast, the structurally related cyclopentadienylidene (CP), a highly electrophilic and reactive carbene, engages the surface through both its HOMO and LUMO, resulting in a tilted adsorption geometry and adsorption energies nearly twice those of NHCs. CP and diphenylcarbene 1 have similar electronic structures and reactivity in the gas phase and in solution: both are highly reactive triplet ground-state carbenes with singlet states that exhibit both strong electrophilicity and nucleophilicity.^9^ In the gas phase, diphenylcarbene 1 adopts a three-dimensional conformation in which both phenyl rings are twisted to relieve steric congestion while maintaining significant LUMO delocalisation. On the surface, the symmetry is broken: one phenyl ring of 1 couples more strongly to the surface than the other.^10^ As a result, relative to CP, the LUMO of diphenylcarbene 1 is lowered in energy by surface interactions, which might result in enhanced electrophilicity.

**Scheme 1:**
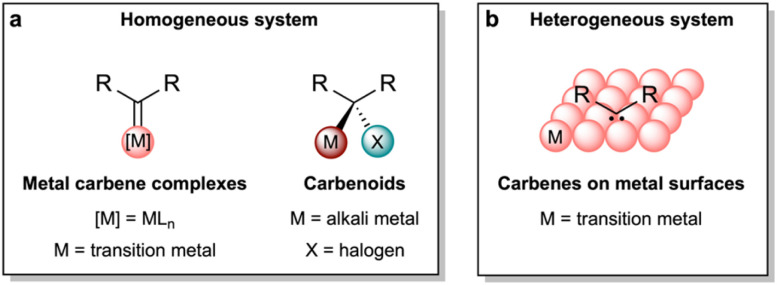
Schematic representation of (a) metal carbene complexes (left) and carbenoids (right) in organometallic chemistry, and (b) carbenes on metal surfaces.

A prerequisite for employing carbenes in synthesis is precise control over their spin state and philicity. In solution and cryogenic matrices, this can be achieved by modulating substituents and tuning the local environment. A striking example is diphenylcarbene 1, where the triplet ground state can be switched to singlet *via* non-covalent interactions with single solvent molecules.^11–13^ Transition metal carbene complexes provide an alternative strategy for controlling carbene reactivity, exemplified by Fischer carbenes with electrophilic carbene centers and Schrock carbenes with nucleophilic centers (left, Scheme 1a).^14^ Moreover, metal carbenoids with a metalated carbon atom that additionally carries a leaving group X, exhibit an ambiphilic character (right, Scheme 1a).^15^ These strategies have been successfully used in homogeneous reactions and catalytic processes.^16–18^

The adsorption of carbenes on metal surfaces provides a complementary strategy for controlling carbene reactivity, with the surfaces functioning as heterogeneous catalysts (Scheme 1b).^19^ As an emerging discipline, on-surface chemistry has revealed that many chemical transformations proceed on surfaces *via* mechanisms that diverge significantly from those in solution.^20–25^ Moreover, several reactive intermediates stabilized by surfaces have been characterized in real space with unprecedented detail using scanning tunneling microscopy (STM) and atomic force microscopy (AFM).^26,27^

Studies of carbene adsorption on metals have largely centered on the formation of ultrastable self-assembled monolayers from persistent carbenes, most notably NHCs.^28–37^ As strong σ donors, NHCs bind strongly to metal surfaces but remain largely unreactive towards other molecules.^32,34,35^ Their stability and commercial accessibility have enabled detailed investigations of their surface binding modes, such as STM tip-induced motion^38^ and patterning^39^ of NHCs. In contrast, comparatively little is known about reactive carbenes, which cannot be isolated and must be generated *in situ* on the surface. These species possess both electrophilic and nucleophilic character and therefore interact with metal substrates *via* combined σ-donor and π-acceptor pathways, resulting in net electron transfer from the metal to the carbenes.^40^ Despite these advances, the fundamental question of how metal surfaces modulate carbene reactivity remains unresolved, even though such insights could enable new classes of surface-mediated catalytic transformations involving carbene intermediates.

**Scheme 2:**
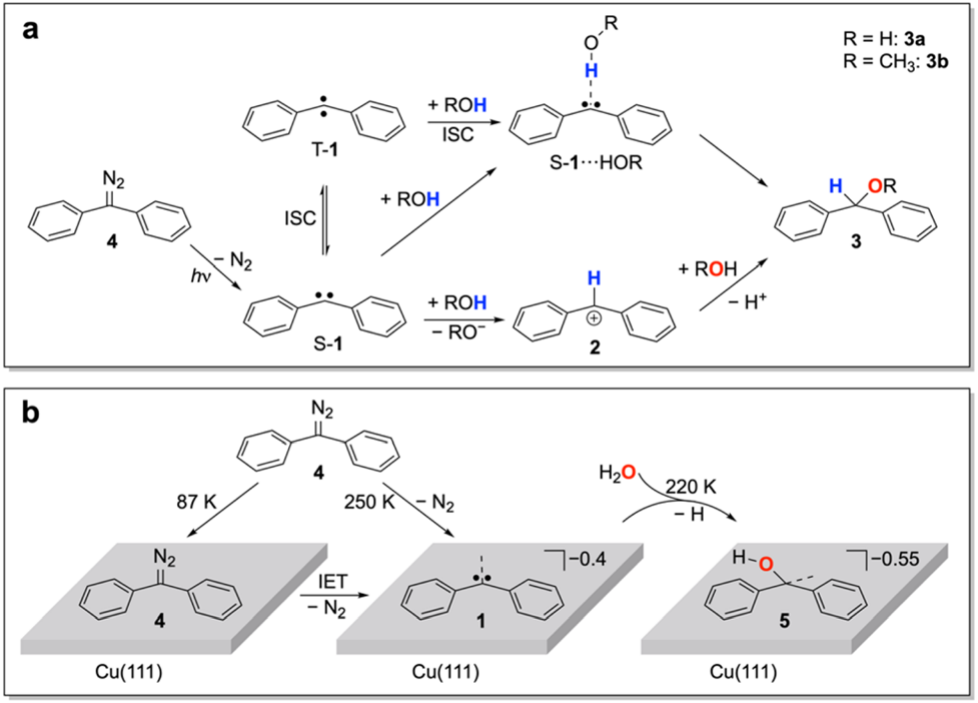
Reaction pathways of diphenylcarbene 1 with water in solutions, cryogenic matrices, and on metal surfaces. (a) Reaction of carbene 1 with water in solutions^6^ and in cryogenic matrices.^13,41^ At minute water concentrations, the complex S-1⋯HOH is the predominant species reacting to diphenylmethanol 3a without the benzhydryl cation 2 intermediate.^13^ At high water concentrations, the cation 2 is the predominant intermediate.^41^ S = singlet state, T = triplet state, ISC = intersystem crossing. (b) Reaction of carbene 1 with water to the new species 5 on Cu(111) surfaces. The numbers indicate the calculated charge on the molecules in electrons.

A prototypical reaction of carbenes is their reaction with water. The mechanism governing the interaction between carbenes such as 1 and water has been debated for decades.^6,13,41–45^ In solutions or in cryogenic matrices, carbene 1 either forms a hydrogen-bonded 1⋯HOR complex,^13^ or undergoes protonation to form the benzhydryl cation 2.^41,43^ Protonation proceeds *via* nucleophilic attack of water or alcohols on 1, and the resulting cation 2 subsequently converts to diphenylmethanol 3a (Scheme 2a).^41^

In this article, we investigate the reactivity of carbene 1 toward water when 1 is generated on a copper surface *via* catalytic dissociation of a diazo precursor 4 (Scheme 2b). Despite its strong binding to the metal, the surface-anchored carbene retains pronounced reactivity, reacting with trace amounts of gas-phase water at temperatures as low as 220 K and exhibiting a remarkably high reaction cross section of 0.6 for a single water collision. In sharp contrast to the nucleophilic pathways observed in solution and cryogenic matrices (Scheme 2a), we show that carbene 1 adsorbed on Cu(111) reacts with water exclusively as an electrophile, yielding the *O*-deprotonated ylide 5 as the sole product (Scheme 2b). Bond-resolved STM provides atomic-scale visualization of the product, and its chemical identity is established through a combination of surface infrared spectroscopy and theoretical modeling.

## Results and discussion

### Formation of carbene 1 from precursor 4 on Cu(111)

Depositing a reactive carbene on a metal surface requires an appropriate precursor. We employ diphenyl diazomethane 4 as a precursor to generate carbene 1 on Cu(111) *via* a catalytic loss of molecular nitrogen.^10^

**Fig. 1:**
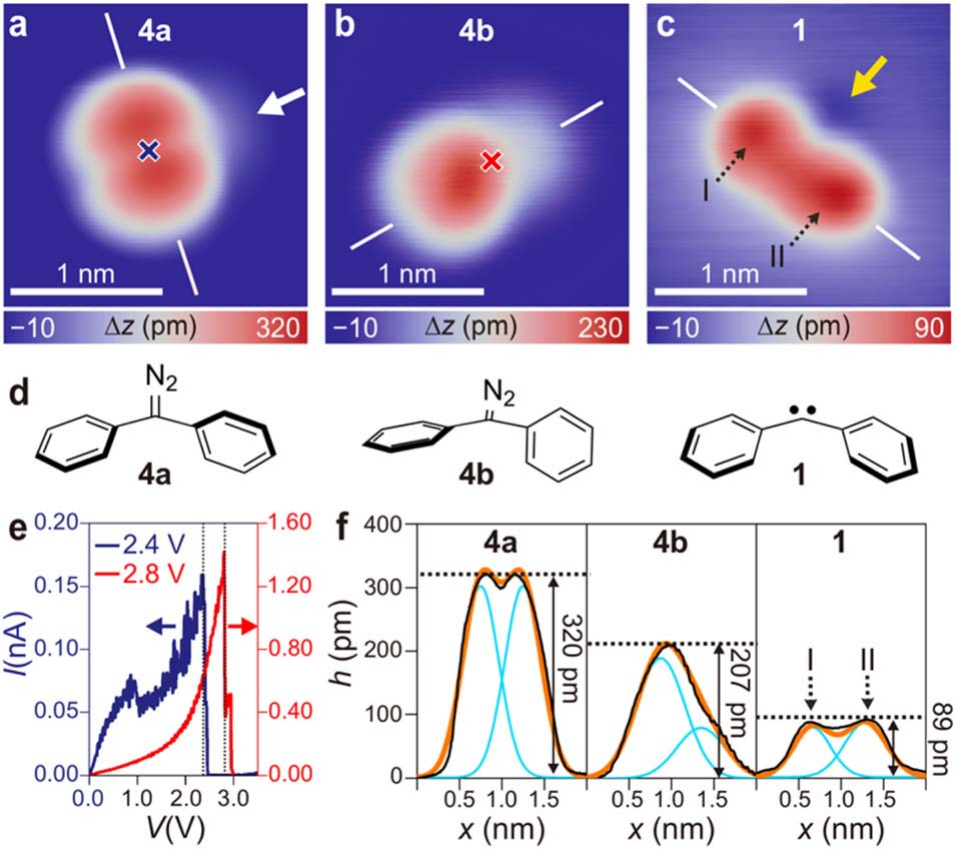
Formation of carbene 1 from precursor 4. STM images of (a and b) precursors: (a) conformer 4a, (b) conformer 4b, and (c) carbene 1 formed by IET manipulation from 4a or 4b. The arrows in (a) and (c) point to a shoulder and a depression, respectively. (d) Schematic models of 4a, 4b, and 1 corresponding to the species in (a to c). (e) *I*–*V* curves while ramping the bias voltage at the crosses in (a, dark blue) and (b, red). (f) Height profiles (black) along lines in (a to c) with double Gaussian fits (cyan) and their sums (orange). I and II in (f) correspond to the protrusions marked in (c). Scanning parameters: (a and b) *V*_b_ = 10 mV, *I*_t_ = 10 pA (c) *V*_b_ = 10 mV, *I*_t_ = 1 nA.

Diphenyl diazomethane 4 adopts a nonplanar structure as revealed by X-ray crystallography,^46^ with rotatable C–C single bonds resulting in considerable conformational flexibility. Upon adsorption on Cu(111) at 87 K, two distinct conformers, 4a and 4b, exist. Conformer 4a (Fig. 1a) is imaged as two elliptical protrusions oriented along the long axis of the molecule and a faint shoulder exclusively at one side (white arrow in Fig. 1a). This appearance of 4a reflects a gas-phase-like structure.^46^ The protrusions in the STM image are assigned to the phenyl rings and the shoulder to the diazo group (Fig. 1d).^47^

In contrast, conformer 4b consists of one elliptical protrusion with a much broader shoulder (Fig. 1b). This feature is consistent with a twisted geometry of 4b arising from an out-of-plane rotation of one of the phenyl rings (Fig. 1d).^47^ Controlled thermal annealing converts 4a to 4b, whereas inelastic electron tunneling (IET) manipulation induces the reverse transformation (Fig. S1), unambiguously confirming that the two species correspond to different conformers of the same molecule.

IET manipulation of precursor 4 also induces an irreversible conversion to a new species (Fig. 1c), as evidenced by sudden drops in the *I–V* spectra (Fig. 1e). The resulting species displays an asymmetric shape consisting of two protrusions that slightly differ in apparent height. The protrusions are assigned to the phenyl rings (dashed arrows I and II in Fig. 1c). Compared to precursor 4, the apparent height of the new species is substantially reduced (Fig. 1f), indicative of a stronger interaction with the Cu(111) surface. In addition, a faint depression appears at the former position of the faint protrusion attributed to the diazo group (yellow arrow in Fig. 1c). The faint depression is interpreted as the formation of a carbene center based on density functional theory (DFT) calculations. The calculations reveal significant charge redistribution upon adsorption of 1 on Cu(111), with a net charge transfer of approximately 0.4*e* from the surface to the carbene.^10^ This behavior is analogous to fluorenylidene on Ag(111), where chemisorption arises from charge transfer from the metal surface to the molecule.^40^ On this basis, we assign the newly formed species to carbene 1 (Fig. 1d), generated by electron–induced cleavage of the C=N bond of 4.

### Reaction of carbene 1 with water

**Fig. 2:**
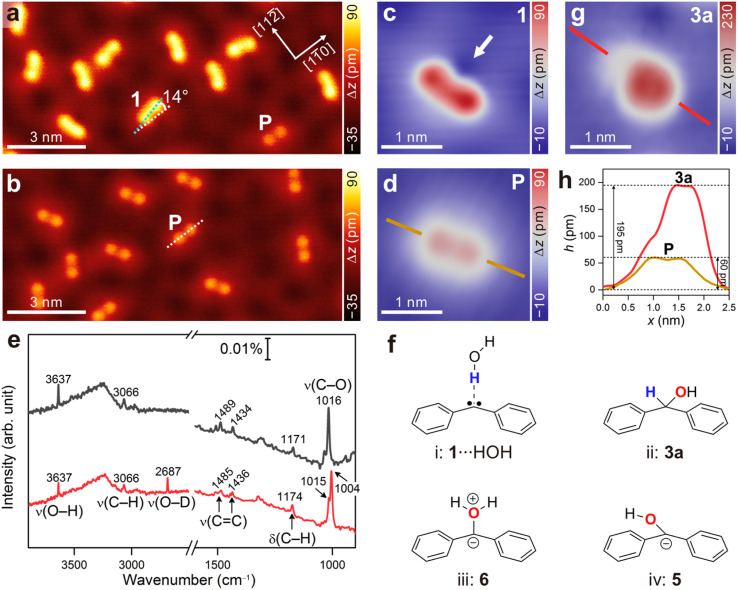
Reaction of carbene 1 with water on Cu(111). (a and b) STM overview images of (a) carbene 1 after deposition of precursor 4 on Cu(111) at 250 K and (b) product P after subsequent exposure to D_2_O at a pressure of 1 × 10^−8^ mbar for 5 min at 220 K. Dashed lines mark the orientations of 1 (cyan) and P (white) on the Cu(111) surface. (c and d) Magnified images of an individual carbene 1 (c) and an individual product P (d) on a false-color scale. The arrow in (c) points to a depression. (e) IR spectra after deposition of 4 at 230 K (black), and 4 + D_2_O at 230 K (red) with wavenumbers. (f) Possible products from the reaction of the carbene 1 with water. (g) STM image of an individual diphenylmethanol 3a on Cu(111). (h) Apparent height profiles of 3a and P along the lines in (d) and (g). Scanning parameters: (a and b) *V*_b_ = 50 mV, *I*_t_ = 5 pA, (c and d) *V*_b_ = 10 mV, *I*_t_ = 1 nA, (g) *V*_b_ = 50 mV, *I*_t_ = 10 pA.

IET manipulation produces only a limited number of carbene 1 molecules. Since the dissociation of diazo groups in precursors such as 4 is catalyzed by copper (Doyle–Kirmse reaction),^48,49^ we examine at which temperature 4 undergoes thermal dissociation during its adsorption on the copper surface. At low-temperature adsorption (87 K), 4 remains intact (Fig. 1a and b). In contrast, adsorption at 250 K predominantly yields molecules with the shape, apparent height, asymmetry, and orientation of carbene 1 (Fig. 2a and c, *cf.*, Fig. 1c), indicating efficient thermal dissociation of 4 at this temperature. Additionally, trace amounts of a new species are observed (P in Fig. 2a), which are not formed *via* IET-induced dissociation of 4. This suggests that a small fraction of carbene 1 already reacts with residuals to form P even under ultrahigh vacuum (UHV) conditions (1.6 × 10^−10^ mbar in the preparation chamber; for details, see Methods). Product P is imaged as two symmetric protrusions displaying an approximately 30% lower apparent height than the carbene 1 (Fig. 2c and d), suggesting a different interaction with Cu(111). Moreover, the orientations of P and 1 towards the 〈110〉 surface directions differ, indicating different adsorption geometries of these two species on Cu(111) (Fig. S2). P is oriented along one of the Cu 〈110〉 directions while 1 derivates by an angle of ± (14 ± 2)° from it (Fig. 2a and b and S2). Given the extraordinarily high reactivity of carbene 1 toward water,^6^ we propose that it reacts with trace water from the residual gas, even at the low partial water pressure of only 5 × 10^−11^ mbar.

To test this proposition, we co-adsorb water to preadsorbed carbene 1 at 220 K. At this temperature, the water adsorbs only transiently on pristine Cu(111).^50^ Thus, a reaction requires a direct collision of water from the gas phase with carbene 1. After exposure to 1 × 10^−8^ mbar water for 5 min, all carbene 1 molecules were converted to product P (Fig. 2b).

The chemical identity of product P is probed by surface infrared reflection absorption spectroscopy (IRRAS). Formation of carbene 1 in the presence of residual H_2_O in the vacuum chamber (see Methods) gives rise to a distinct O–H stretching vibration peak at 3637 cm^−1^ (black curve in Fig. 2e). Co-adsorption of D_2_O onto preadsorbed carbene 1 produces a corresponding O–D stretching vibration at 2687 cm^−1^, accompanied by a concomitant decrease in the O–H signal from residual H_2_O (red curve in Fig. 2e). The resulting OH/OD isotopic shift of 1.35 is consistent with typical values reported for related systems, including CH_3_OH/CH_3_OD (1.35) and HOD (1.36) in the gas phase,^51^ as well as H_2_O/D_2_O and OH/OD species on surfaces (1.36 and 1.35, respectively).^52^ These IR experiments unequivocally confirm the reaction of carbene 1 with water. Moreover, the IR activity of the O–H/O–D stretching mode indicates that the bond is tilted relative to the surface plane, in accordance with the IR surface selection rule, which requires a nonzero projection of the dynamic dipole moment along the surface normal.^53^

The O–H/O–D stretching vibrations could arise either from an OH/OD group attached to the carbene or from hydroxyl groups generated *via* carbene-catalyzed H_2_O/D_2_O dissociation. However, the absence of depressions in the STM images rules out the presence of isolated hydroxyl groups.^54,55^ Several other possible structures for P are considered (Fig. 2f): (i) an H-bonded complex S-1⋯HOH, (ii) diphenylmethanol 3a, (iii) an oxonium-ylide 6, and (iv) an *O*-deprotonated ylide 5. To assign P we combine STM manipulations, IR spectra, and on-surface DFT calculations. (i) An H-bonded complex S-1⋯HOH is inconsistent with the IR spectrum. The O–H stretching vibration of P at 3637 cm^−1^ corresponds to an O–H stretching vibration of an OH group rather than a hydrogen-bonded water molecule. The O–H stretching vibration of S-1⋯HOH isolated in rare gas matrices has a much higher frequency of 3697 cm^−1^ (for details, see Fig. S3).^41^ This assignment is further supported by STM manipulation, as P remains intact during IET manipulation and scanning-induced motion or rotation, consistent with a covalently bonded species rather than an H-bonded complex (Fig. S4). (ii) Diphenylmethanol 3a is ruled out as P by sub-monolayer deposition on Cu(111) (Fig. S5). Individual molecules 3a appear as elongated pumpkin-shaped protrusions with a shoulder, indicating a nonplanar structure (Fig. 2g), in contrast to the planar structure of P with its two symmetric protrusions (Fig. 2d). Additionally, the apparent height of 3a is more than three times that of P at the same bias voltage (Fig. 2h; see Fig. S5 for details). IR spectroscopy provides a chemical characterization of 3a. The IR spectrum of 3a differs from that of P, confirming that they are distinct species (Fig. S5). (iii) Ylide 6 possesses two O–H stretching modes, symmetric and asymmetric, inconsistent with a single sharp O–H stretching mode of P in the IR spectra (Fig. 2e). DFT calculations indicate that ylide 6 is unstable on Cu(111) and dissociates into surface-bonded 5 upon optimization (Fig. S6). Although 6 may transiently exist in the gas phase, it is not stabilized on the surface.

Based on these results, we assign P to 5, the *O*-deprotonated ylide (Fig. 2f). The formation of the C–O bond in 5 is confirmed by H_2_O/D_2_O isotopic experiments. The vibrational band at 1016 cm^−1^, corresponding to the C–O stretching mode of 5, shifts to 1004 cm^−1^ after D_2_O exposure, while other vibrational features (*e.g.*, C–H stretching and C=C stretching modes) remain unchanged (Fig. 2). The observed redshift is in perfect agreement with the calculated isotopic shift of 12 cm^−1^ based on the change in reduced mass between C–OH and C–OD.

The formation of 5 requires that, following the collision of a water molecule with carbene 1, the complex undergoes deprotonation or dehydrogenation.

### Geometry of *O*-deprotonated ylide 5 on Cu(111)

**Fig. 3:**
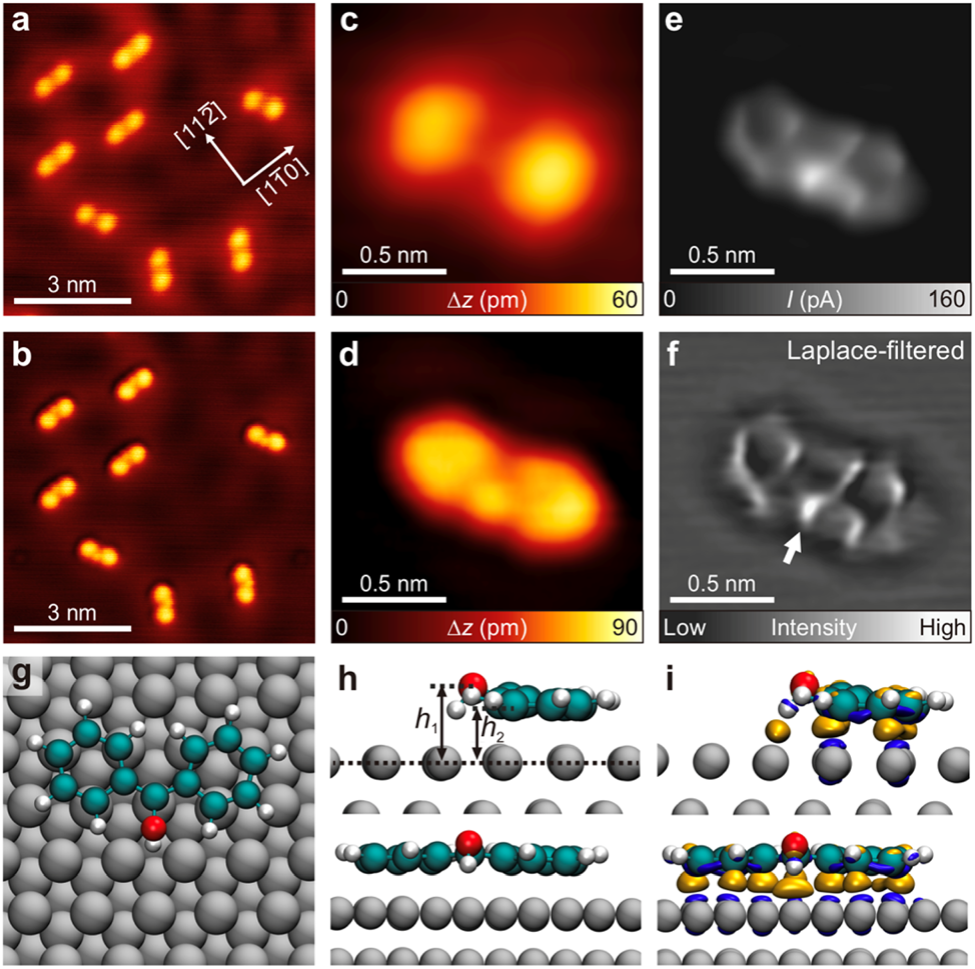
Geometry of 5 on Cu(111). (a and b) STM overview images of 5 on Cu(111) imaged with (a) a metallic tip and (b) a functionalized tip. (c to e) Magnified images of an individual 5 imaged with a metallic tip (c) and a functionalized tip (d and e); (c and d) are recorded in constant current mode and (e) in constant height mode. (f) Laplace-filtered image of bond-resolved STM image (e). White arrow: see text. Scanning parameters: *I*_t_ = 10 pA, and (a and c) *V*_b_ = −10 mV, (b) *V*_b_ = −100 mV, (d) *V*_b_ = 5 mV, (e) *V*_b_ = 5 mV, Δ*z* = + 50 pm. (g and h) Optimized geometry of 5 on Cu(111) in (g) top view and (h) side views in two orientations. Grey spheres: copper; dark cyan spheres: carbon; red spheres: oxygen; white spheres: hydrogen. *h*_1_ and *h*_2_ in (h) mark the distances from the surface plane to the oxygen atom of the OH group and from the surface plane to the closer phenyl ring, respectively. (i) Charge density difference between the combined system, 5 and Cu(111), and the individual components with the positions of the atoms in adsorbate and surface as in the adsorbed system. Yellow and blue regions indicate charge accumulation and depletion, respectively (see Methods for details).

The geometry of 5 on Cu(111) is elucidate by bond-resolved STM imaging. A metallic tip images 5 as two lobes that indicate the positions of the phenyl rings without resolving the position of the hydroxyl group (Fig. 3a and c). To enhance lateral resolution, the tip is functionalized^56,57^ by picking up another molecule 5 (Fig. S8), revealing an extra feature between the lobes (Fig. 3b and d). This feature can be attributed either to the central carbon atom or to the attached hydroxyl group.

Imaging 5 with this functionalized tip at closer tip-molecule distances within the Pauli repulsion region^58,59^ leads to a bond-resolved STM image (Fig. 3e). The two slightly tilted hexagonal phenyls of the molecular backbone are clearly resolved, including the intramolecular C–C bonds connecting the central carbon atom and the phenyl groups, even better visible in the Laplace-filtered image (Fig. 3f). Notably, the central spot appears elongated perpendicular to the long axis of the molecule, providing evidence for an additional group bonded to the central carbon atom (white arrow in Fig. 3f). Its higher contrast relative to the phenyl rings indicates a smaller tip-adsorbate separation, consistent with a moiety protruding further from the surface than the phenyl rings (for details, see Fig. S9).

The experimental observations are corroborated by DFT calculations. For the structurally optimized *O*-deprotonated ylide 5 on the Cu(111) surface, the interaction of the central carbon atom with Cu(111) is no longer dominated by direct bonding to the surface (Fig. 3g and h). This is in contrast to carbene 1, where the interaction with the surface resembles a formal C–metal bond,^10^ whereas for 5 the interaction with the surface is less localized. The optimized structure shows 5 adsorbed with both phenyl rings nearly parallel to the surface (Fig. 3h), consistent with the two symmetric protrusions observed in our STM images (Fig. 2c). Moreover, 5 aligns along one of the Cu〈110〉 directions, in agreement with the STM experiments, whereas carbene 1 is calculated to adsorb at an angle of 20° relative to one of the 〈110〉 directions,^10^ consistent with the experimentally observed angle of (14 ± 2)° (see Fig. S2 for a definition of the angles).

Importantly, the O–H bond of 5 is not parallel to the surface (Fig. 3h). The predicted angle between the surface plane and the vector defined by the O–H bond in the optimized structure is 54°. This is in agreement with the IR spectroscopic evidence discussed above. The calculated height of the oxygen atom of the OH group of 5 is 70 pm greater than that of the closest phenyl carbon atom (*h*_1_ = 276 pm *vs. h*_2_ = 208 pm, Fig. 3h), placing it closer to the STM tip. This vertical displacement accounts for the enhanced contrast of the central feature in the bond-resolved STM image (Fig. 3f), demonstrating full consistency between theory and experiment. Overall, these results establish that the Cu(111) surface catalyzes the formation of a product that is inaccessible under conventional solution-phase or matrix-isolation conditions.^6,41^

To understand the stability of 5 on the surface, we examine the charge redistribution of an adsorbed 5. Charge accumulation occurs in the region between 5 and the surface, as visualized in Fig. 3i by the charge density differences. The charge density of the copper atoms directly interacting with 5 decreases (blue in Fig. 3i) due to the charge transfer from the surface to molecule 5, while concomitant charge accumulation appears between 5 and the copper surface (yellow). The out-of-plane orientation of the hydroxyl group of 5 is influenced by this charge redistribution, directing the orientation of the rotatable O–H bond with respect to the oxygen atom. The resulting orientation of the hydroxyl group, with the oxygen atom pointing towards the vacuum, accounts for the IR intensity of the O–H and O–D stretching peaks of 5 (Fig. 2e), in accordance with the IR surface selection rule.^53^ Furthermore, 5 is stabilized through surface-mediated charge compensation to become the final product. Under exposure to excess water (1 × 10^−8^ mbar for 5 min at 220 K) 5 does not further react to form diphenylmethanol 3a. This behavior contrasts with the *O*-deprotonated ylide 5 in solution, which is short-lived and readily reacts with water to yield diphenylmethanol 3a, as predicted by quantum mechanics/molecular mechanics (QM/MM) molecular dynamics (MD) simulations.^7^

Calculated atomic charges of 5 on Cu(111) indicate that there is a net charge transfer from the surface to the molecule of approximately 0.5*e*. The calculated charge distribution of surface-bonded 5 is compared to that of 5 in the gas phase, modeled as a formal cation, anion, or radical in the same geometry of the adsorbate (Fig. S7). The charges at the OH group and at the carbon atom bonded to it resemble those of the formal radical species, whereas the charges at the remaining carbon atoms display charge values closer to the formal anion. Consequently, 5 adsorbed on Cu(111) can be described as an intermediate between a radical and an anion.

We next examine the catalytic role of Cu(111) in enabling the high reactivity of carbene 1. Even during the deposition of precursor 4 at 250 K, a small fraction of 1 reacts with the residual water from the UHV background pressure to form the *O*-deprotonated ylide 5 (Fig. S10). From titration experiments exposing 1 on Cu(111) to residual water at 220 K we estimate a remarkably large cross-section of ∼0.6 per single collision (Fig. S10). This value is orders of magnitude greater than those reported for other surface reactions involving water, such as the hydrogenation of graphene nanoribbon edges by water on Au(111) with a cross-section below 2 × 10^−3^ at room temperature.^60^ Since water adsorbs only transiently on Cu(111) at 220 K,^50^ the reaction proceeds *via* a direct collision of gas phase water with the carbene molecule, consistent with an Eley–Rideal-type mechanism.

A detailed computational investigation of the Eley–Rideal-type mechanism^61^ would be computationally highly demanding for our system within DFT. Instead, we rationalize the high reactivity of carbene 1 based on its interaction with the copper surface. In the gas phase, in solution, and in cryogenic matrices, carbene reactivity is largely determined by its spin state.^62,63^ Carbene 1 has a triplet ground state with a small S–T gap, which can be switched to singlet *via* hydrogen or halogen bonding.^11–13^ On metal surfaces, however, the spin state of carbene 1 is ill-defined due to the electron transfer from the surface, as evidenced by a calculated charge difference map showing substantial charge redistribution between 1 and the Cu(111) surface (Fig. S11). Unlike stable NHCs, which act as pure σ donors,^64^ carbene 1 receives a net electron transfer from the copper surface, exhibiting characteristics of both Schrock- and Fischer-type carbenes. Surface-adsorbed carbene 1 resembles a Schrock carbene, showing charge transfer from the metal to the carbene center. Yet, it is electrophilic towards water, similar to a Fischer carbene. Nevertheless, carbenes on metal surfaces differ fundamentally from metal carbene complexes for two reasons: (i) the extended metallic surface acts as an electron reservoir, allowing greater flexibility in tuning charge transfer than a single metal center, and (ii) surface-adsorbed carbenes are ligand-free, whereas metal carbene complexes contain additional coordinating ligands.

Lateral STM manipulation moves carbene 1 across the surface *via* noncovalent tip-induced interactions (Fig. S12). It indicates a moderate strength of the C–Cu interaction, which facilitates the reaction of the carbene center with water and explains the remarkably high reactivity of 1 on Cu(111), with a reaction cross-section of 0.6 even at 220 K. The formation of 5 catalyzed by a Cu(111) surface is remarkable because neither time-resolved absorption spectroscopy in solution^6,7^ nor low-temperature spectroscopy in cryogenic matrices^41^ has provided evidence for 5 as an intermediate in a reaction between carbene 1 and water. Singlet carbene 1 is one of the strongest neutral bases,^7^ and consequently undergoes ultrafast protonation in protic solvents.^6,7,65^ Matrix isolation experiments demonstrated that 1 reacts with water and protic solvents exclusively *via* protonation, rather than as an electrophile.^13,41^ At low water concentrations, the hydrogen-bonded complex S-1⋯HOH predominates and reacts to diphenylmethanol 3a without involvement of the benzhydryl cation 2.^13^ At high water concentrations, cation 2 becomes the primary intermediate.^41^ Similarly, reactions of 1 with methanol in solutions or cryogenic matrices proceed *via* proton transfer to generate the short-lived benzhydryl cation 2 (Scheme 2a).^43^ Although the vacant p orbital of carbene 1 could, in principle, allow electrophilic attack on heteroatom lone pairs, only QM/MM molecular dynamics simulations suggest a minor electrophilic pathway involving two water molecules.^7^

Here, we provide the first experimental evidence that carbene 1 exhibits electrophilic reactivity toward water when catalyzed by a copper surface. This finding reveals a previously inaccessible reaction pathway and highlights the catalytic potential of metal surfaces to modulate carbene reactivity.

## Conclusions

By combining IRRAS and bond-resolved STM to study the co-adsorption of 1 with water on Cu(111), we demonstrate that the metal surface catalyzes the formation of *O*-deprotonated ylide 5. Surface-adsorbed carbene 1 reacts as a highly efficient electrophile with gas-phase water molecules below room temperature, yielding this previously unobserved species. The metal surface directs the reaction between precursor 4 and water in multiple ways: (i) it catalyzes the dissociation of precursor 4 into N_2_ and carbene 1, (ii) it immobilizes the carbene 1 (ref. 10) while retaining its reactivity, and (iii) it stabilizes the charges on the *O*-deprotonated ylide 5 and the proton *via* charge transfer. In this manner, the metal surface mitigates charge build-up during heterolytic bond cleavage. While metal carbene complexes are extensively exploited in homogeneous catalysis, our results establish that adsorption on metal surfaces enables novel reaction pathways for carbenes in heterogeneous environments. This approach may also be extended to other highly reactive intermediates – such as nitrenes, phosphinidenes, silylenes, and borylenes – which exhibit context-dependent nucleophilic or electrophilic behavior. Adsorption on metal surfaces thus provides a versatile strategy to steer the reactivity of a broad family of reactive intermediates in tailored surface environments.

## Author contributions

Y. C., E. S. G., W. S., and K. M. conceptualized the project. Y. C. performed the STM and IR measurements. J. F. R. synthesized the precursor molecules. J. M. P. performed the calculations. All the authors contributed to the data interpretation and writing the manuscript.

## Conflicts of interest

There are no conflicts to declare.

## Supplementary Material

SC-017-D6SC00488A-s001

## Data Availability

The data supporting the findings of this study are available within the article and its supplementary information (SI). Supplementary information: methods, supporting STM data, computational results, and synthetic procedures of chemical compounds reported in this study. See DOI: https://doi.org/10.1039/d6sc00488a.
