# Supplementary material for: Electrophilic activation of water by a carbene catalyzed by a copper surface
Source: Chem Sci. 2026 Jun 6. Online ahead of print. doi: 10.1039/d6sc00488a (PMC13267005; doi:10.1039/d6sc00488a)
Supplement: Supplementary file 1 [file SC-OLF-D6SC00488A-s001.pdf]

Supplementary Information (SI) for Chemical Science.
This journal is © The Royal Society of Chemistry 2026

Supporting Information

Electrophilic activation of water by a carbene catalyzed by a copper surface

Yunjun Cao[1], Joel Mieres-Perez[2], Julien Frederic Rowen[3], Elsa Sanchez-Garcia[2],*

Wolfram Sander[3], and Karina Morgenstern[1]**

[1]Physical Chemistry I, Ruhr-Universität Bochum, D-44801 Bochum, Germany

[2]Computational Bioengineering, Technische Universität Dortmund, D-44227 Dortmund, Germany

[3]Organic Chemistry II, Ruhr-Universität Bochum, D-44801 Bochum, Germany

*Corresponding authors: elsa.sanchez@tu-dortmund.de (E.S.G.); wolfram.sander@rub.de (W.S.); karina.morgenstern@rub.de (K.M.)

Contents

1. Synthesis of chemical compounds 2
2. Methods 4
3. Isomerization and dissociation of precursor **4** 7
4. Orientations of carbene **1** and product **P** on Cu(111) 8
5. Vibrational frequencies of hydroxyl groups and molecular water 9
6. Covalent bond of product **P** 11
7. Diphenylmethanol **3a** vs. product **P** on Cu(111) 12
8. Oxonium-ylide **6** on Cu(111) 14
9. Bond resolution of O-deprotonated ylide **5** 15
10. Reaction of carbene **1** with residual water from UHV background 17
11. Charge density difference map of carbene **1** on Cu(111) 18
12. Scanning-induced motion of carbene **1** on Cu(111) 18
13. References 19

1. Synthesis of chemical compounds

General: The chemicals were used as received without further purification. Aluminum oxide (neutral, Brockmann Activity I) was deactivated by adding water to reach Brockmann Activity IV one day prior to its use as stationary material for column chromatography. NMR spectra were recorded on a Bruker Neo-400 MHz spectrometer. The chemical shifts are given in ppm referenced to DMSO-d_6 (^{1}H NMR: 2.5 ppm).

Synthesis:

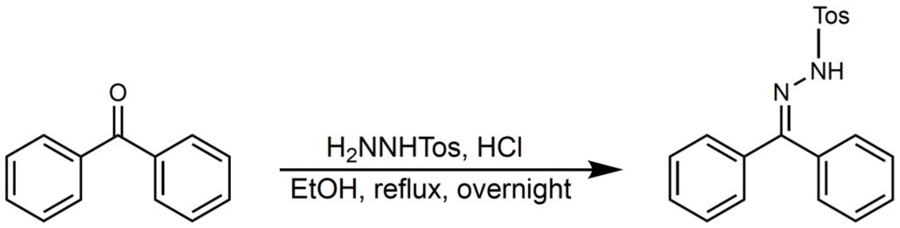

Following a literature procedure,[1] 2.0 g (11 mmol) of benzophenone (99%, Carl Roth) and 4.22 g (22.7 mmol) of *p*-toluenesulfonyl hydrazide (97%, Sigma-Aldrich) were suspended in 60 mL of ethanol (99.8 %, Sigma-Aldrich) and 1 mL of conc. HCl (37%, VWR) was added under stirring. The suspension was refluxed overnight. After cooling down, the precipitate was filtrated, washed with small amounts of ethanol, and dried *in vacuo*. Purification was achieved by recrystallization from ethyl acetate (HPLC grade, VWR). 2.37 g (6.8 mmol, 61.8 %) of benzophenone tosylhydrazone were obtained as colorless crystals.

^{1}H NMR (400 MHz, DMSO-d_6): δ/ppm = 10.44 (s, 1H), 7.83 (d, J = 8.3 Hz, 2H), 7.56 – 7.48 (m, 3H), 7.43 (d, J = 8.1 Hz, 2H), 7.38 – 7.30 (m, 3H), 7.28 – 7.20 (m, 4H), 2.39 (s, 3H).

^{13}C NMR (100 MHz, DMSO-d_6): δ/ppm = 155.02, 143.82, 137.64, 136.56, 133.05, 130.17, 129.91, 129.85, 129.29, 128.82, 128.24,127.70, 21.53.

The compound was characterized by comparison of its NMR spectra with published data.[1]

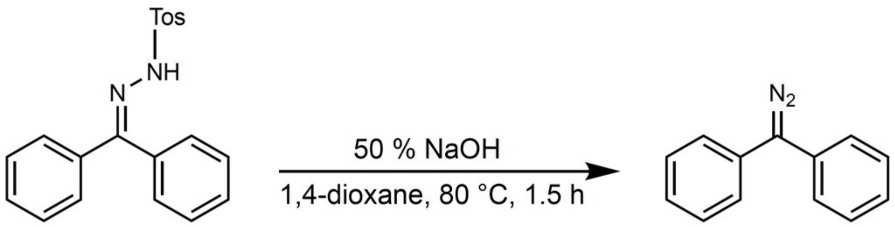

Following a literature procedure,[2, 3] 2.0 g (5.7 mmol) of benzophenone tosylhydrazone was suspended in 25 mL of 1,4-dioxane (p.A., Fisher Scientific) and 3 mL of 50% aq. NaOH solution (NaOH pellets, Sigma-Aldrich) was added. The suspension was stirred vigorously at 80°C for 1.5 h. After cooling to room temperature, 20 mL of distilled water was added, the organic phase separated, and the aqueous phase extracted three times with 20 mL n-pentane (99%, VWR) until it was colorless. The combined organic phases were washed two times with 50 mL of distilled water, dried over $MgSO_4$ (99%, Carl Roth), and evaporated *in vacuo*. Purification was achieved by column chromatography (deactivated aluminum oxide (neutral, Acros Organics, Brockmann Activity IV); n-pentane as eluent). 390 mg (2.0 mmol, 35.2 %) of diphenyl diazomethane **4** were obtained as a purple solid.

^{1}H NMR (400 MHz, DMSO-d_6): δ/ppm = 7.49 – 7.40 (m, 4H), 7.33 – 7.26 (m, 4H), 7.24 (tt, *J* = 7.0, 1.2 Hz, 2H).

^{13}C NMR (100 MHz, DMSO-d_6): δ/ppm = 129.35, 128.53, 125.83, 124.83.

The compound was characterized by comparison of its NMR spectra with published data.[3]

2. Methods

Experimental details. The experiments were performed in two UHV systems with similar facilities for sample preparation and molecule deposition. The first system is equipped with a low-temperature STM (Unisoku) operated at 5.1 K (base pressure below 1.0 × 10^{-10} mbar), a preparation chamber (base pressure below 1.6 × 10^{-10} mbar), and a sealed-off molecule deposition chamber (base pressure below 4.0 × 10^{-10} mbar); the second with a vacuum Fourier transform infrared spectroscopy (FTIR) spectrometer (Bruker, VERTEX 80V), an IR chamber (base pressure below 6.0 × 10^{-10} mbar), and a sealed-off molecule deposition chamber (base pressure below 1.0 × 10^{-8} mbar).[4] The molecule deposition chambers are separated from the preparation chamber (STM system) or the IR chamber (IR system) by a gate valve.

Sample preparation. The Cu(111) and Au(111) surfaces were cleaned by standard cycles of sputtering and annealing. The parameters used for Cu(111) in the STM system are sputtering with 1.0 keV Ne^+ at 3.4 × 10^{-5} mbar yielding 1 µA for 30 min and annealing at 900 K for 10 min, and for Cu(111) and Au(111) in the IR system sputtering with 1.3 keV Ne^+ at 3.0 × 10^{-5} mbar yielding 10 µA for 30 min and annealing at 850 K for 10 min.

Molecules deposition. For precursor **4** deposition, freshly prepared **4** was transferred in an argon atmosphere into a UHV-cleaned quartz tube sealed by an angle valve called a molecule unit. After loading the molecules, the molecule unit was attached to the molecule deposition chamber. The quartz tube was immersed into a cooling bath with an ethanol/LN_2 mixture, except during deposition constantly kept below 253 K, to avoid dissociation because of its thermal instability. **4** was further purified by freeze-pump-thaw cycles, using a mass spectrometer to check the purity of its vapor. Before deposition, the filaments were switched off to prevent dissociation and fragment formation.

The bare Cu(111) surface was placed either in the preparation chamber of the STM system or in the IR chamber of the IR system during the molecule dosing of **4** through the respective molecule deposition chambers. Yet, the pressure at the surface is by orders of magnitudes lower than the pressure measured in the molecule deposition chamber. In the STM system, **4** was deposited at a surface temperature of 87 K or 250 K for 133 s at a pressure of 3.2 × 10^{-7} mbar in the molecule deposition chamber. In the IR system, **4** was

deposited at a surface temperature of 230 K for 120 s at a pressure of 3.5×10^{-7} mbar in the molecule deposition chamber.

Diphenylmethanol **3a** (99%, Sigma Aldrich) was transferred to a molecule unit. It was purified by pumping at room temperature for several days. **3a** was deposited at a surface temperature of 85 K for 135 s at a pressure of 2.2×10^{-7} mbar in the molecule deposition chamber. The **3a** covered surface was annealed at 220 K for 133 s before the transfer to the STM head.

D_2O of milli-Q quality was further purified in vacuum through freeze-pump-thaw cycles. In the STM system, the chamber was flushed by D_2O to reduce the partial pressure of H_2O prior to transferring the sample into the preparation chamber. Note that the IR chamber cannot be flushed by D_2O prior to deposition because the measured spectra are obtained by subtracting a background spectrum. Any sample transfer after recording the background spectrum causes artifacts in the spectra. Consequently, an order of magnitude higher partial H_2O pressure in the IR chamber (about 2.2×10^{-10} mbar) and its molecule deposition chamber (5.1×10^{-9} mbar) than in the STM preparation chamber causes a reaction of **1** even without D_2O exposure.

In the STM system, a D_2O pressure of 5.0×10^{-7} mbar was set in the molecule deposition chamber for deposition on the **4**-covered surface at 220 K for 300 s. The corresponding pressure of the preparation chamber is 1.0×10^{-8} mbar. In the IR system, a partial pressure of 3.5×10^{-6} mbar was set in the molecule deposition chamber at a ratio of **4** : D_2O = 1 : 9 for co-adsorption of **4** with D_2O. This molecule mixture was subsequently deposited for 120 s at a surface temperature of 230 K.

STM measurements. STM images were obtained with a Pt/Ir tip. The bias voltage was applied to the sample. For inelastic electron tunneling (IET) manipulation, the tip was positioned above a chosen part of the molecule before the feedback loop was switched off. A constant voltage was applied while recording the current-time ($I - t$) trace, or the voltage was ramped from zero to a predefined value while recording the current-voltage ($I - V$) spectra. A step-like change in the tunneling current indicated a successful manipulation, verified in a subsequent STM image. The orientation of the absorbed molecules was defined

as the angle between a line through the center of both protrusions in the STM images and the <110> directions of the surface.

IRRAS measurements. The IRRAS measurements were performed in reflection absorption mode at a fixed incidence angle of 80°. The optic bench was evacuated to eliminate absorption from gas-phase species (e.g., H_2O, CO, and CO_2) in the optical path. The infrared reflection absorption spectra were obtained by subtracting a background spectrum recorded before molecule exposure. The IR spectra represent an average of 1024 to 2048 scans at a 4 cm^{-1} resolution.

Computational details. The calculations were performed with the Quantum-ESPRESSO package.[5] The metal surface was simulated with a copper slab comprising four layers of Cu(111) using (6 × 6) atoms in each layer. The bottom two layers were kept fixed at the value of the experimental lattice constant. The rest of the structure was optimized. The atomic simulation environment (ASE)[6] was used to construct the slabs. The PBEsol functional was used for all calculations, employing D3 dispersion corrections to consider the van der Waals interactions.[7, 8] A combination of ultrasoft and plane-augmented wave pseudopotentials with a wavefunction cut-off of 55 Ry and a charge density cut-off of 660 Ry, was used. The Brillouin zone was sampled using the gamma point only algorithm, as implemented in Quantum-ESPRESSO. The adsorption distances were calculated as the difference in the *z* position values between the adsorbed atoms and the surface atom directly below the oxygen atom or the average of values from the closest surface atoms when no copper atom was directly below the adsorbed atom (carbon atom from the ring closer to the surface). The Bader charges were obtained using the Critic2 software[9, 10] using density calculated with QUANTUM ESPRESSO. The charge transfer distribution was plotted (Fig. 3i) by subtracting the charge distribution of the organic species and the surface (in the same geometry as in the complex) from the charge distribution of the complex. The charge distribution of the organic species was computed using single point calculations assuming the organic species as neutral. Charge distribution calculations depend on how the atoms are defined within the molecule. We use the charges calculated using the AIM (Atoms in Molecules) formalism, which have delivered results consistent with the experimental trends for similar systems.[11, 12]

3. Isomerization and dissociation of precursor 4

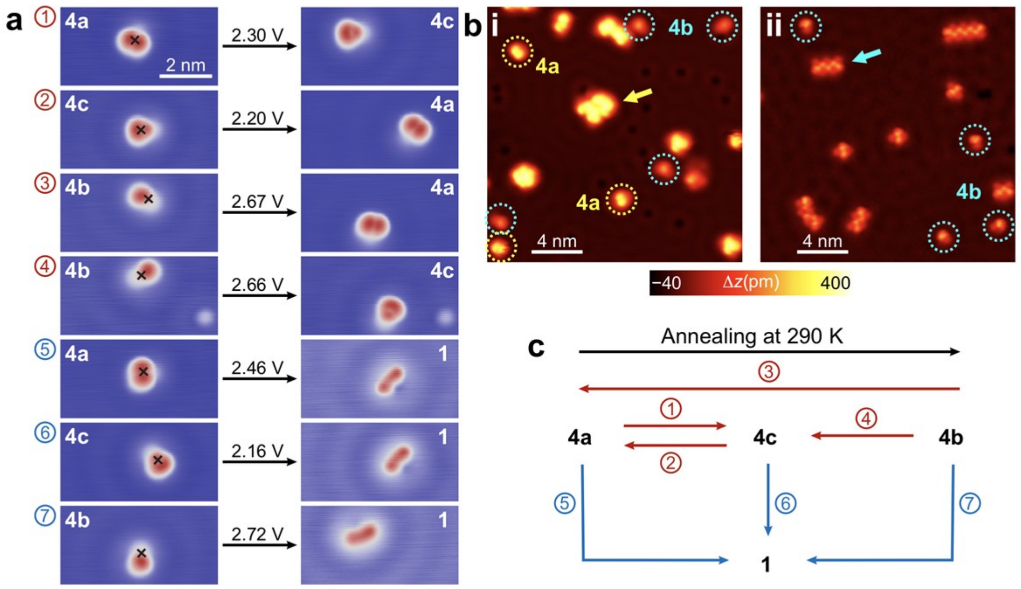

Fig. S1. Isomerization and dissociation of precursor **4**. (a) Series of STM images before (left) and after (right) IET manipulation that alters the molecule at the given voltage. The IET manipulations were performed by ramping the bias voltage at the crosses (left) from 0.01 V to 3.00 V at a step size of 0.01 V and a dwelling time of 50 ms for each step. (b) STM overview images of **4** deposited on Cu(111) at 87 K (i) and subsequent annealing at 290 K (ii). Yellow and cyan circles mark isolated **4a** and **4b**. Yellow and cyan arrows point to a cluster of **4a** and a chain of **4b**, respectively. Scanning parameters: (a) V_b = 100 mV, I_t = 10 pA (b-i) V_b = 200 mV, I_t = 10 pA (b-ii) V_b = 100 mV, I_t = 5 pA. (c) Scheme of the switching and dissociation events of **4** as observed in (a) and (b).

In the main text (Fig. 1), we explored the dissociation of precursors **4a** and **4b** to diphenylcarbene **1**. Here, we demonstrate via IET manipulation that **4a** and **4b** are conformers. First, **4a** is changed to an intermediate **4c** (step 1 in Fig. S1a), which displays two protrusions of different heights. The **4c** intermediate is changed back to **4a** (step 2 in Fig. S1a). Furthermore, **4b** is changed to **4a** or **4c** (steps 3 and 4 in Fig. S1a). In addition, **4a** is changed to **4b** upon annealing at 290 K (Fig. S1b). Therefore, **4a**, **4b**, and **4c** are reversible switches, indicating that they are conformers (Fig. S1c). The **4a**, **4b**, and **4c** conformers are assigned to twisted geometries with different dihedral angles, which are stabilized by the molecule-surface interactions. Finally, the **4a**, **4b**, and **4c** precursors are irreversibly converted to the novel molecule **1** (steps 5 to 7 in Fig. S1a), suggesting the formation of carbenes.

4. Orientations of carbene 1 and product P on Cu(111)

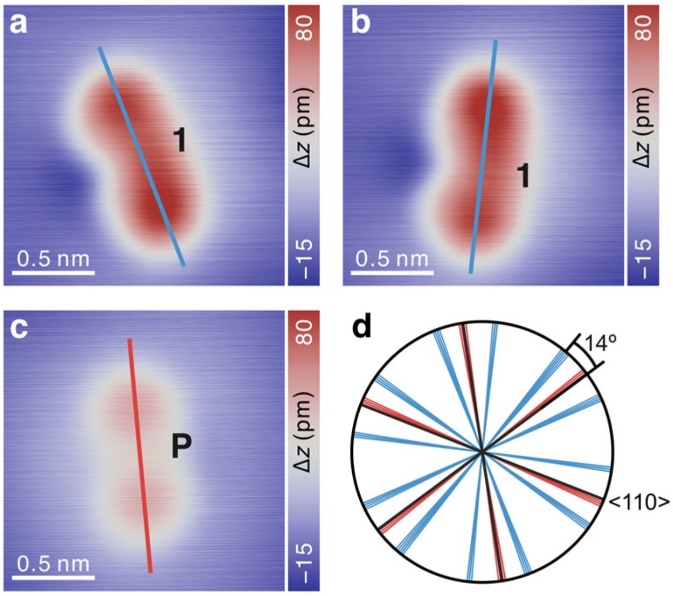

Fig. S2. Orientations of carbene **1** and product **P** on Cu(111). (a-c) STM images of (a, b) carbene **1** and (c) the product **P** on Cu(111). Blue and red lines in (a, b) and (c) are drawn across both protrusions. (d) Angular plot of the orientations of **1** (blue lines) and the **P** (red lines). Black lines mark the <110> directions of the Cu(111) surface. The plot is based on N(**1**) = 73 and N(**P**) = 21. Scanning parameters: (a–c) V_b = 10 mV and I_t = 1 nA.

In the main text, we revealed that the newly formed product **P** and the carbene **1** differ in their apparent height (Fig. 2c,d). Here, we demonstrate that the two species also differ in their orientations. The curved shape of **1** introduces a chirality on Cu(111). The right-handed (Fig. S2a) and the left-handed (Fig. S2b) conformers cannot be superimposed on each other by rotation or translation.[13] The two **1** conformers are rotated by ± (14 ± 2)° to the <110> directions of Cu(111) (blue lines in Fig. S2d). In contrast, the product **P** (Fig. S2c) is non-chiral. Its only conformer is oriented along the <110> directions of the surface (red lines in Fig. S2d). The different orientations of the carbene **1** and the product **P** corroborate that these are distinct species.

5. Vibrational frequencies of hydroxyl groups and molecular water

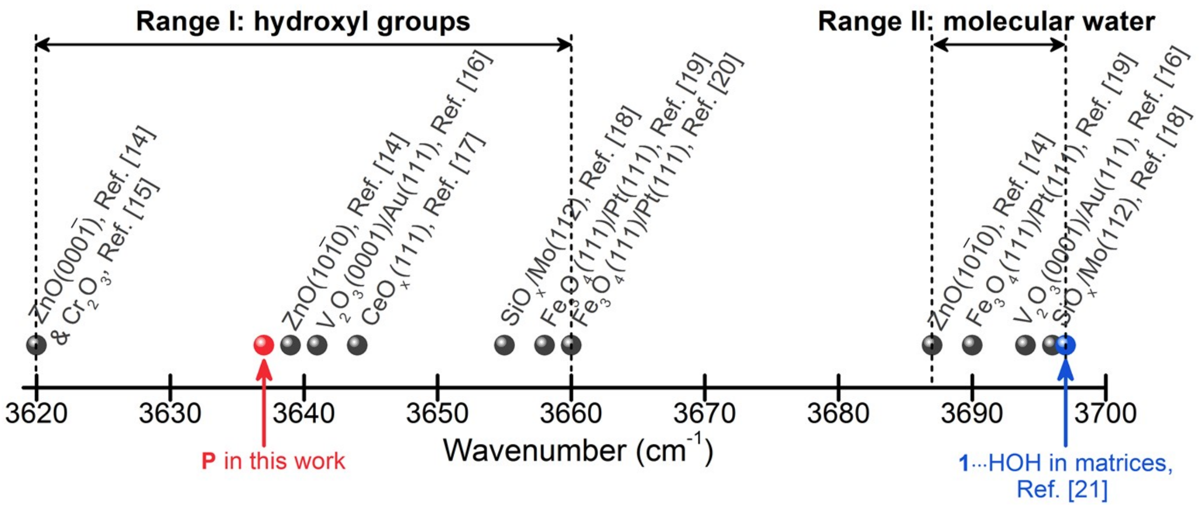

Fig. S3. Vibrational frequencies of surface-supported hydroxyl groups and molecular water. Vibrational frequencies for molecular water on surfaces or in cryogenic matrices and hydroxyls from dissociation of water on surfaces. Corresponding frequency values are listed in Table S1.

Table S1. Vibrations in IR spectra for hydroxyl groups and molecular water on various surfaces and in cryogenic matrices.

Surfaces&matrices	Hydroxyl OH (OD) (cm^{-1})	Molecular H_2O (D_2O) (cm^{-1})	References
ZnO(000$\bar{1}$)	3620 (2669)		[14]
Cr_2O_3	3620		[15]
ZnO(10$\bar{1}$0)	3639 (2683)	3687 (2716)	[14]
V_2O_3(0001)/Au(111)	3641	3694	[16]
CeO_x(111)	3644 (2688)		[17]
SiO_x/Mo(112)	3655	3696	[18]
Fe_3O_4(111)/Pt(111)	3658, 3660	3690	[19, 20]
**1**···HOH in matrices		3697	[21]
**P** in this work	3637 (2687)		

In the main text, we deduce that the product **P** comprises a hydroxyl group (Fig. 2e). Here, we exclude the possibility that **P** is a H-bonded **1**···HOH complex based on its vibrational frequency.

Generally, a hydroxyl group has a lower O–H stretching frequency than molecular water. Molecular water in gas phase has a symmetric stretching vibration at 3657 cm^{-1} and an asymmetric stretching vibration at 3756 cm^{-1}.[22] Fig. S3 and Table S1 summarize the vibrational frequencies of hydroxyl groups and molecular water on various surfaces. On metal surfaces, water usually adorbs molecularly. Because of its low diffusion barrier, water monomers aggregate to hydrogen-bonded clusters.[23] Such a hydrogen-bonded network substantially reduces the stretching frequencies of water from that of gas-phase water. For example, the stretching frequencies of water reduce to 3460 cm^{-1} and 3383 cm^{-1} on Cu(110)[24] and to 3390 cm^{-1} on Ru(0001).[25] Frequencies in this range do not exist in our case. Therefore, we compare the frequency of **P** to that on metal oxide surfaces[14-20] (Fig. S3 and Table S1). Hydroxyl groups and molecular water often coexist on metal oxide surfaces, facilitating a direct comparison of their vibrational frequencies. The O–H stretching frequencies of hydroxyl groups range from 3620 cm^{-1} to 3660 cm^{-1} (range I in Fig. S3). The O–H stretching frequencies of molecular water cover 3687 cm^{-1} to 3697 cm^{-1} (range II in Fig. S3). A gap of approx. 40 cm^{-1} clearly separates the two frequency ranges.

The O–H stretching mode of product **P** at 3637 cm^{-1} points to a hydroxyl group (range I in Fig. S3). In contrast, the O–H stretching mode of **1**···HOH isolated in rare gas matrices[21] at 3697 cm^{-1} is consistent with molecular water (range II in Fig. S3). Therefore, the frequency comparison proves that **P** is not a **1**···HOH complex.

6. Covalent bond of product P

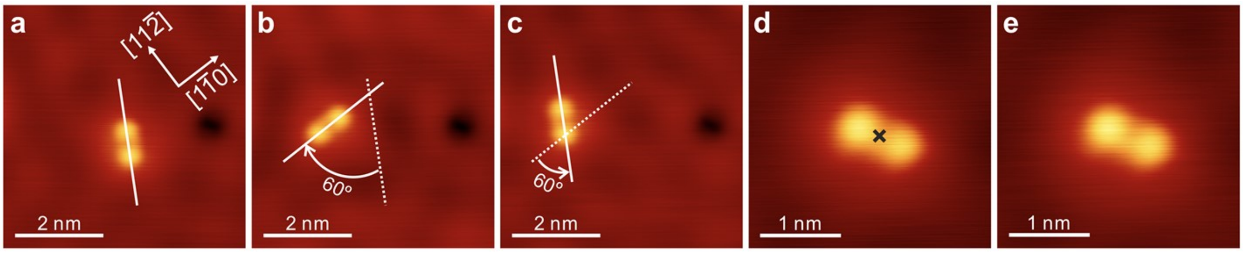

Fig. S4. Manipulation of product **P**. (a–c) STM images of sequential scanning-induced motion and rotation of **P**. The scanning-induced motion and rotation were performed by scanning the same region in constant height mode at V_b = 10 mV, I_t = 1 nA, and Δz = + 200 pm. Positive values of Δz represent a closer tip-surface distance than the setpoint. Successful motion and rotation were verified in subsequently scanning in constant current mode at V_b = 10 mV, I_t = 1 nA, and Δz = 0 pm. Solid white lines mark the long axis of the molecule. Dotted white lines mark the long axis of the molecule in the previous image before the scanning-induced motion and rotation. (d, e) STM images (d) before and (e) after injection of electrons into **P** for 10 s at a bias voltage of 2.0 V and a tunneling current of 1.2 nA. The cross in (d) marks the injection site.

In Section 5, we deduced from a frequency analysis that the product **P** is unlikely a **1**···HOH complex. Here, we further corroborate this point by STM manipulation.

First, **P** remains intact during the scanning-induced motion and rotation (from Fig. S4a to S4c). The rotation of 60° for **P** is consistent with its angular distribution, i.e., along the surface <110> directions (Fig. S6d). Second, the hydrogen bond in the **1**···HOH complex (Fig. 2f) should be separable through the injection of inelastic electrons above a threshold energy far below that necessary for covalent bond scission. For example, the cleavage of hydrogen bonds within water clusters is feasible by injecting electrons with energies between 0.15 eV and 0.51 eV.[26, 27] Above 1.6 eV, the water molecules within water clusters were dissociated on Cu(111).[27] Here, the product **P** is not altered by injection of electrons up to 2.0 eV (Fig. S4d to 4e). The manipulation experiments corroborate that the product **P** is a covalently-bonded species rather than a **1**···HOH complex.

7. Diphenylmethanol 3a vs. product P on Cu(111)

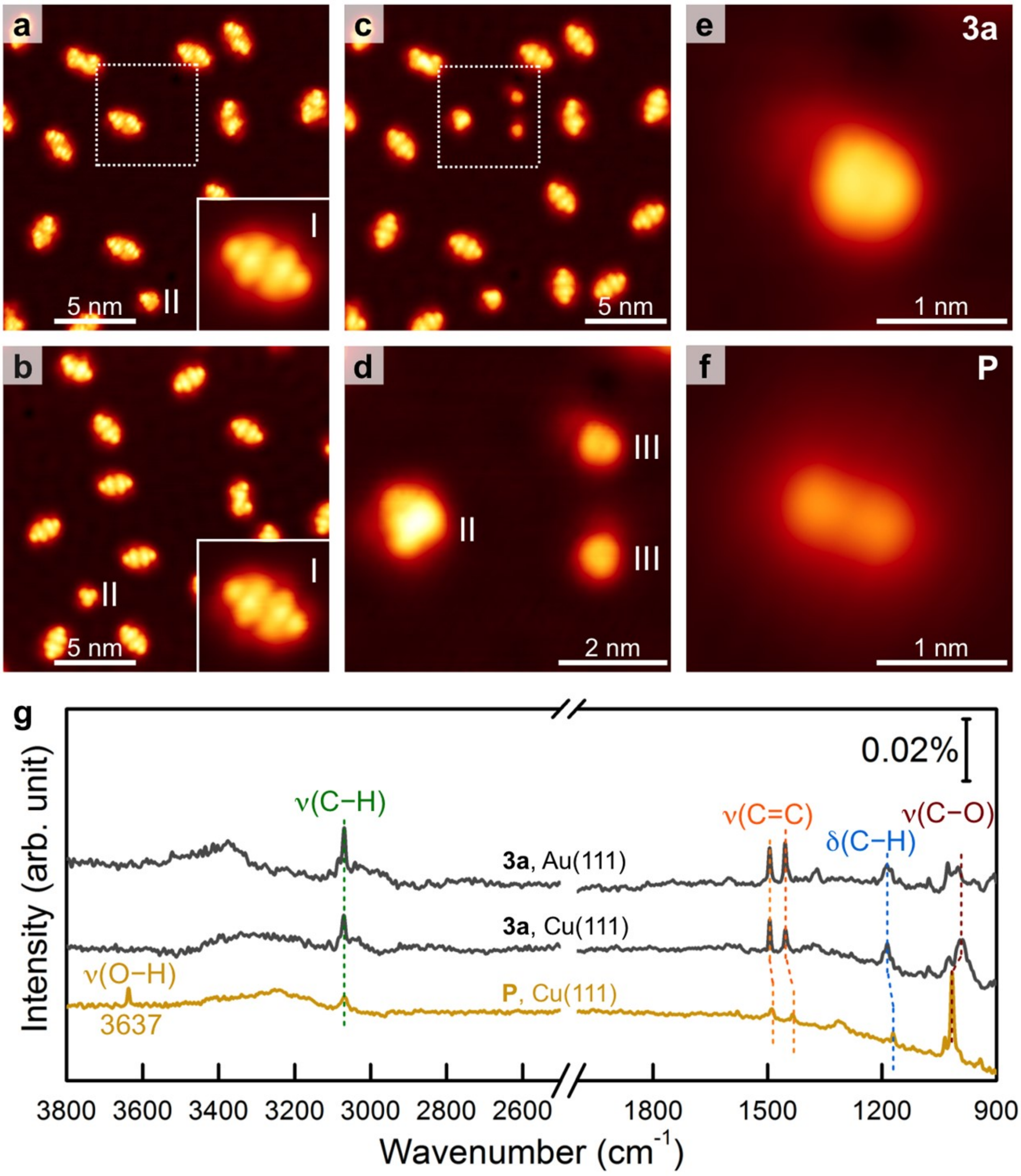

Fig. S5. Diphenylmethanol **3a** vs. product **P** on Cu(111). (a,b) STM overview images of diphenylmethanol **3a** on Cu(111) after annealing at 220 K (a) and after subsequent exposure to D_2O (1×10^{-8} mbar, 5 mins) at the same temperature (b). Insets: magnified STM images of **3a** tetramers (3 nm × 3 nm). (c) Disassembled cluster in the dashed square (a) by a series of lateral manipulations in constant current mode at a tunneling resistance of 625 kΩ. (d) Magnified image of the square in (c). (e, f) STM images of (e) **3a** and (f) **P**. Scanning parameters: (a-e) V_b = 50 mV, I_t = 5 pA, (f) V_b = 50 mV, I_t = 10 pA. (g) IR spectra of **3a** (black curves) adsorbed on Cu(111) and Au(111), and product **P** (dark yellow curve) on Cu(111). **3a** was deposited at 230 K for 10 s at a pressure of 3.6×10^{-6} mbar.

In the main text, several possible **P** structures are considered (Fig. 2f). We rule out the structure of diphenylmethanol **3a** for **P** by depositing **3a** in sub-monolayer coverage on Cu(111). After adsorption at 85 K and annealing at 220 K, **3a** self-assembles into monosized tetragonal clusters (I in Fig. S5a), with very few exceptions (II in Fig. S5a). Exposure to D_2O under the conditions used for the reaction of **1** to **P** does not alter the structure (Fig. S5b). Through lateral manipulations, we disassemble a tetragonal cluster into three parts (Fig. S5c). The larger part (II in Fig. S5d) is approximately half the size of the tetragonal cluster, while the two smaller parts (III in Fig. S5d) correspond to **3a** monomers based on their size. **3a** and **P** differ in apparent heights. At the same bias voltage of 50 mV, the apparent height of **3a** is more than three times that of **P** (Fig. 2h), indicating they are different species. The **3a** monomer is imaged as an elongated, pumpkin-shaped protrusion with a shoulder (Fig. S5e), indicating a non-planar structure, in contrast to the planar structure of **P**.

IR spectroscopy provides a chemical characterization of **3a**. The IR spectrum of **3a** differs clearly from that of **P**, as evidenced by frequency shifts of the C=C stretching, C−H deformation, and C−O stretching modes (Fig. S5g). The O−H stretching region around 3600 cm^{-1} is featureless for **3a** on Cu(111). To exclude the possibility that **3a** is dehydrogenated on Cu(111), we deposited **3a** on the chemically inert Au(111) surface. The signals of O−H stretching modes are also absent (Fig. S5g). The absence of an O−H mode can be explained by the IR surface selection rule. Modes vibrating exclusively in the surface plane are IR inactive. Consequently, it indicates that the O−H bond is adsorbed parallel to the surface. Importantly, the vibrations of **3a** on Cu(111) and Au(111) are very similar, but differ from those of **P**, confirming that **3a** and **P** are distinct species.

8. Oxonium-ylide 6 on Cu(111)

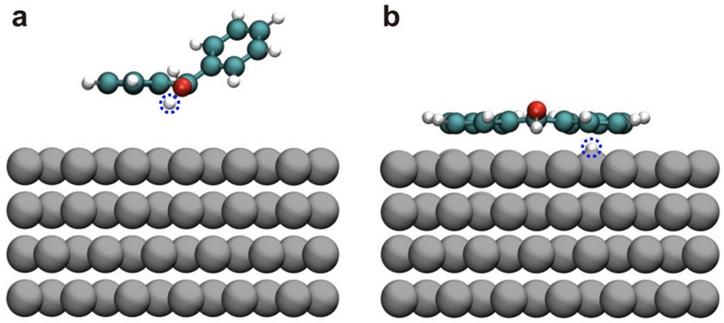

Fig. S6. Oxonium-ylide **6** on Cu(111). (a) Initial structure for oxonium-ylide **6** optimization on Cu(111). The ylide **6** was placed at a distance of 0.3 nm to the surface. (b) Optimized structure – the optimization converges to **5**. The hydrogen abstracted by the surface is marked by a dashed blue circle.

To exclude that the product **P** in the main text is an oxonium ylide (Fig. 2f), we optimized the ylide **6** on Cu(111). **6** was placed at a distance of 0.3 nm to the surface with the structure in the gas phase (Fig. S6a). During the optimization, **5** was formed from the ylide **6** (Fig. S6b). Following the reasoning of Sections 5, 6, and 7 and the calculations in this section, we assign the product **P** to **5**, a O-deprotonated ylide.

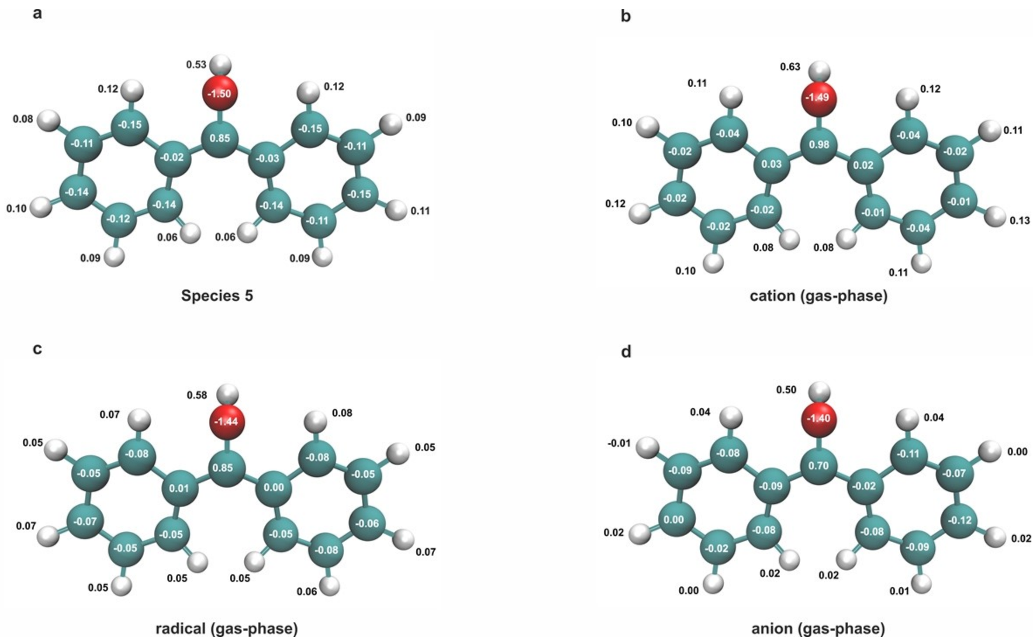

Fig. S7. Calculated atomic charges of **5**: (a) on Cu(111) and (b-d) as formal cation, radical and anion in gas phase in the same geometry as on Cu(111). The surface atoms have been removed for clarity. Dark cyan spheres: carbon atoms; red sphere: oxygen atom; white spheres: hydrogen atoms.

9. Bond resolution of O-deprotonated ylide 5

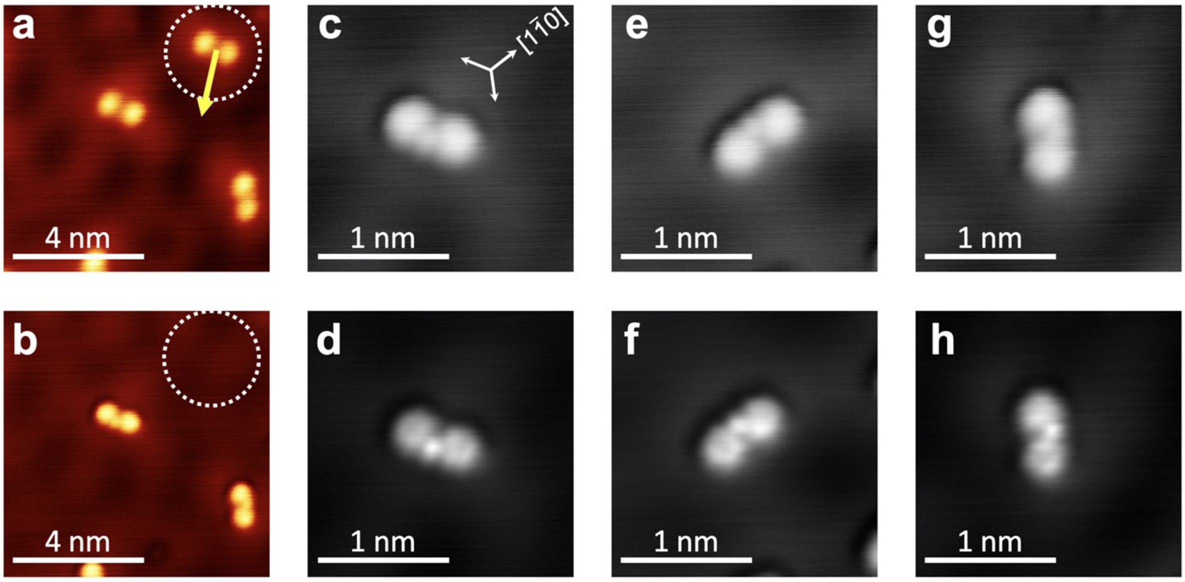

Fig. S8. Tip modification for bond resolution of **5**. (a, b) STM images (a) before and (b) after picking up a molecule **5** (marked by white circles) by lateral manipulation along the yellow arrow at a tunneling resistance of R = 0.25 MΩ. (c-h) STM images of **5** in three different orientations recorded by the functionalized tip in (c, e, g) constant current mode and (d, f, h) constant height mode. Scanning parameters and setpoint: I_t = 10 pA and (a) V_b = – 10 mV (b) V_b = –100 mV (c-h) V_b = – 5 mV.

The STM image of **5** recorded with a metallic tip consists of two lobes only, neither allowing us to identify the position of its carbon center nor its relative height to the side lobes. The STM resolution is enhanced by transferring a molecule from the surface to the tip via lateral manipulation. The success of such a transfer is verified by imaging the same area before and after manipulation (Fig. S8a to 8b). The enhanced contrast resolves an extra spot between the two lobes (Fig. S8b). It is excluded that this additional spot is a tip artefact by the identical appearance of **5** in three different orientations (Fig. S8c,e,g).

Recording images with the functionalized tip in constant height mode resolves the bonds of **5** (Fig. S8d,f,h). Moreover, the relative heights/intensities of the central spot and two side lobes are reversed from between constant current (Fig. S8c,e,g) and constant height mode (Fig. S8d,f,h), demonstrating a higher height of the hydroxyl group above the surface than the phenyl rings.

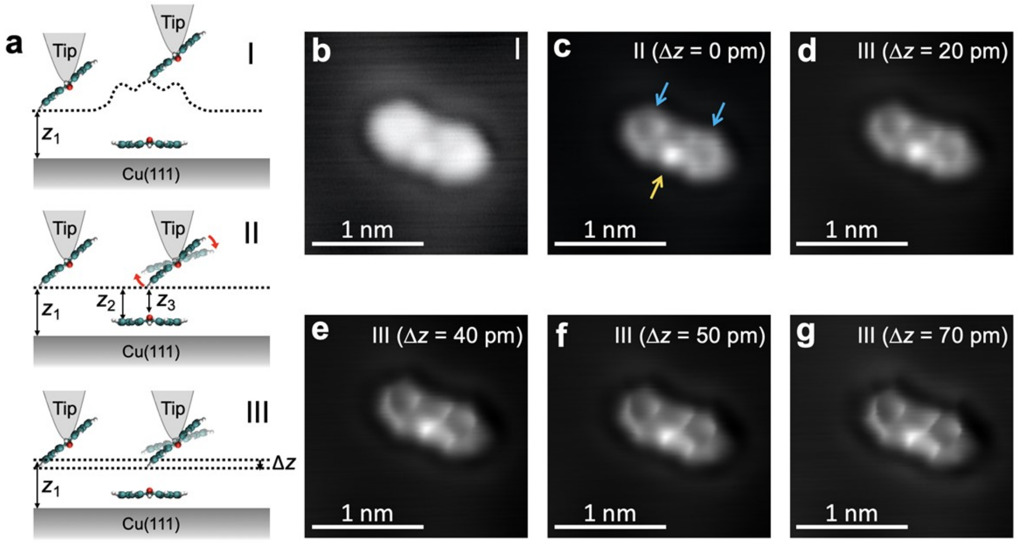

Fig. S9. Height-dependent bond-resolved STM images. (a) Schematics of the origin of the contrast difference in constant current and constant height modes. Dotted lines display the tip height during the scanning motion. I. Constant current mode scan; z_1 is the starting tip-Cu(111) distance. II. Constant height mode scan: z_1 is the original tip-Cu(111) distance. z_2 and z_3 mark the tip-phenyl and tip-hydroxyl distances. III: Constant height mode scans: ($z_1 - \Delta z$) is a reduced tip-Cu(111) distance. (b) STM image of **5** recorded by the functionalized tip in constant current mode. (c–g) Bond-resolved STM images of the same molecule **5** recorded by the functionalized tip in constant height mode. Here, z_1 is determined by a setpoint of V_b = 5 mV and I_t = 10 pA. The scan was performed by approaching the tip from z_1 to surface by a distance of (c) 0 pm, (d) 20 pm, (e) 40 pm, (f) 50 pm, and (g) 70 pm.

In Fig. 3f of the main text, an elongated feature vertical to the molecular long axis confirms a side group bonded to the central carbon atom (white arrow). Here, we discuss the higher contrast of the side group (hydroxyl group) than the phenyl groups in the bond-resolved STM images.

In constant current mode, the STM image is a convolution of its real height and the local density-of-states (LDOS) of adsorbates, such that the real adsorbate height cannot be determined straightforwardly (Fig. S9a-I). Though the imaging mechanism is not yet understood completely, it is established that the tunneling current is altered for bond-resolved STM images in response to the force of the molecule acting on a flexible tip.[28] This force changes marginally in constant current mode, where the tip is mainly at a constant distance from the molecule. In constant height mode, the tip-adsorbate distance is smaller

when the tip is above the adsorbate, increasing the force of the adsorbate on the tip. The force contribution to the tunnelling current is sensitive to the tip-adsorbate distance, reflecting the relative heights of different functional groups, tip-phenyl and tip-hydroxyl, if the tip reaches the Pauli repulsion region (Fig. S9-II). Therefore, the higher contrast of the hydroxyl group (yellow arrow in Fig. S9c) than the phenyl groups (blue arrows in Fig. S9c) in the bond-resolved STM image points to a higher geometric height. Furthermore, approaching the tip closer to the surface (Fig. S9-III) improves the resolution, while the relative contrast of the hydroxyl group to the phenyl groups persists (Fig. S9d-g). As stated in the main text, the higher contrast of the hydroxyl group than of the phenyl groups (Fig. 3f) indicates a non-planar geometry of the hydroxyl group to the surface (Fig. 3g), which agrees with the sharp O–H and O–D stretching vibrations of **5** in the IR spectra (Fig. 2e).

10. Reaction of carbene 1 with residual water from UHV background

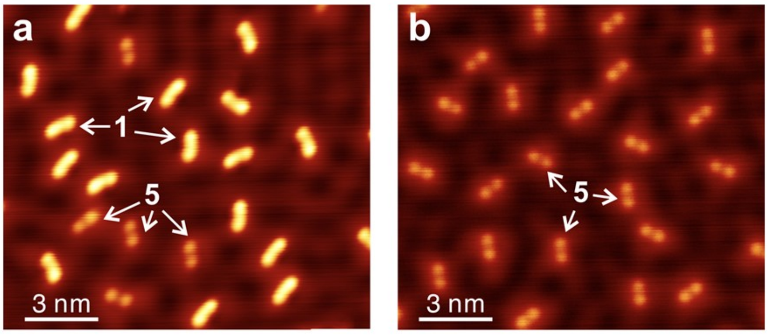

Fig. S10. Reaction of carbene **1** with residual water from UHV background. STM images of (a) as grown **1** on Cu(111) at 250 K and (b) after exposure to the UHV background of the preparation chamber. Scanning parameters: V_b = 10 mV, and (a) I_t = 1 nA , (b) I_t = 10 pA.

As discussed in the main text, the reactivity of **1** on Cu(111) with water is extraordinarily high. Consequently, a small number of carbene **1** already converted to **5** during the deposition of the precursor **4** at 250 K and transfer time of the sample from the preparation chamber into the cold shields around the STM (Fig. S10a). The reasoning is supported by a long-time exposure (13 hours) of the **1**-covered Cu(111) to the UHV background (base pressure of 1.6 × 10^{-10} mbar). During that time, **1** reacted fully to **5** (Fig. S10b). We determine a cross-section of ~0.6 for a single water collision to **1** based on the partial water pressure (5 × 10^{-11} mbar) and exposure time.

11. Charge density difference map of carbene 1 on Cu(111)

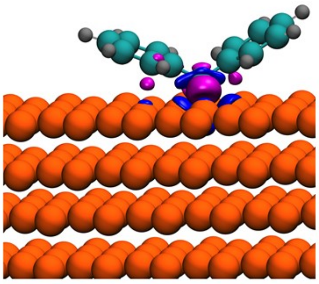

Fig. S11. Charge density difference between the combined system, carbene **1** and Cu(111), and the individual components with the positions of the atoms in adsorbate and surface as in the adsorbed system.[13] Blue and pink regions represent charge depletion and charge accumulation, respectively. The isosurface value is $4 \cdot 10^{-3}\, e^{-}\, \mathrm{bohr}^{-3}$.

In the main text, we mentioned that carbene **1** has a triplet ground state and a small S-T gap in the gas phase and cryogenic matrices. On metal surfaces, the spin state of carbenes cannot be defined due to the charge transfer from the surface to the carbene. It is evidenced by the calculated charge difference map of **1** on Cu(111), which reveals a charge redistribution and electron transfer between **1** and the surface (Fig. S11).

12. Scanning-induced motion of carbene 1 on Cu(111)

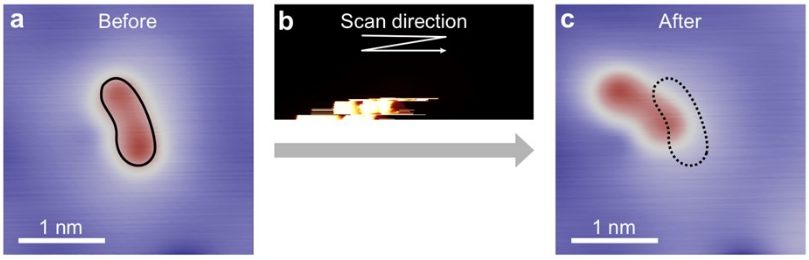

Fig. S12. STM images of scanning-induced motion of **1** on Cu(111). (a) before, (b) during, and (c) after scan-induced motion. The motion was induced by scanning the region in (b) in constant height mode at a setpoint of V_b = 5 mV and I_t = 1 nA on the Cu(111) surface. Solid contour marks the initial position of **1** and dotted contour marks the position of **1** in the previous frame. Scanning parameters: (a, c) V_b = 10 mV, I_t = 1 nA.

13. References

1. P.-L. Wu, S.-Y. Peng and J. Magrath, Tosylhydrazines by the reduction of tosylhydrazones with triethylsilane in trifluoroacetic acid, *Synthesis*, 1996, **1996**, 249-252.
2. A. Jonczyk and J. Wlostowska, A simple method for generation of diazocompounds in an aqueous two-phase system, *Synth. Commun.*, 1978, **8**, 569-572.
3. M. Lee, Z. Ren, D. G. Musaev and H. M. L. Davies, Rhodium-stabilized diarylcarbenes behaving as donor/acceptor carbenes, *ACS Catal.*, 2020, **10**, 6240-6247.
4. Y. Wang, A. Glenz, M. Muhler and C. Wöll, A new dual-purpose ultrahigh vacuum infrared spectroscopy apparatus optimized for grazing-incidence reflection as well as for transmission geometries, *Rev. Sci. Instrum.*, 2009, **80**, 113108.
5. P. Giannozzi, O. Andreussi, T. Brumme, O. Bunau, M. Buongiorno Nardelli, M. Calandra, R. Car, C. Cavazzoni, D. Ceresoli, M. Cococcioni, N. Colonna, I. Carnimeo, A. Dal Corso, S. de Gironcoli, P. Delugas, R. A. DiStasio, A. Ferretti, A. Floris, G. Fratesi, G. Fugallo, R. Gebauer, U. Gerstmann, F. Giustino, T. Gorni, J. Jia, M. Kawamura, H. Y. Ko, A. Kokalj, E. Küçükbenli, M. Lazzeri, M. Marsili, N. Marzari, F. Mauri, N. L. Nguyen, H. V. Nguyen, A. Otero-de-la-Roza, L. Paulatto, S. Poncé, D. Rocca, R. Sabatini, B. Santra, M. Schlipf, A. P. Seitsonen, A. Smogunov, I. Timrov, T. Thonhauser, P. Umari, N. Vast, X. Wu and S. Baroni, Advanced capabilities for materials modelling with Quantum ESPRESSO, *J. Phys.: Condens. Matter*, 2017, **29**, 465901.
6. A. Hjorth Larsen, J. Jorgen Mortensen, J. Blomqvist, I. E. Castelli, R. Christensen, M. Dulak, J. Friis, M. N. Groves, B. Hammer, C. Hargus, E. D. Hermes, P. C. Jennings, P. Bjerre Jensen, J. Kermode, J. R. Kitchin, E. Leonhard Kolsbjerg, J. Kubal, K. Kaasbjerg, S. Lysgaard, J. Bergmann Maronsson, T. Maxson, T. Olsen, L. Pastewka, A. Peterson, C. Rostgaard, J. Schiotz, O. Schutt, M. Strange, K. S. Thygesen, T. Vegge, L. Vilhelmsen, M. Walter, Z. Zeng and K. W. Jacobsen, The atomic simulation environment – a Python library for working with atoms, *J. Phys. Condens. Matter.*, 2017, **29**, 273002.
7. J. P. Perdew, K. Burke and M. Ernzerhof, Generalized gradient approximation made simple, *Phys. Rev. Lett.*, 1996, **77**, 3865-3868.
8. S. Grimme, J. Antony, S. Ehrlich and H. Krieg, A consistent and accurate ab initio parametrization of density functional dispersion correction (DFT-D) for the 94 elements H-Pu, *J. Chem. Phys.*, 2010, **132**, 154104.
9. A. Otero-de-la-Roza, M. A. Blanco, A. M. Pendás and V. Luaña, Critic: a new program for the topological analysis of solid-state electron densities, *Comput. Phys. Commun.*, 2009, **180**, 157-166.

10. A. Otero-de-la-Roza, E. R. Johnson and V. Luaña, Critic2: A program for real-space analysis of quantum chemical interactions in solids, *Comput. Phys. Commun.*, 2014, **185**, 1007-1018.
11. J. Mieres-Perez, K. Lucht, I. Trosien, W. Sander, E. Sanchez-Garcia and K. Morgenstern, Controlling reactivity − real-space imaging of a surface metal carbene, *J. Am. Chem. Soc.*, 2021, **143**, 4653-4660.
12. Y. Cao, J. Mieres-Perez, K. Lucht, I. Ulrich, P. Schweer, E. Sanchez-Garcia, K. Morgenstern and W. Sander, C−C coupling of carbene molecules on a metal surface in the presence of water, *J. Am. Chem. Soc.*, 2023, **145**, 11544-11552.
13. Y. Cao, J. Mieres-Perez, J. F. Rowen, E. Sanchez-Garcia, W. Sander and K. Morgenstern, Chirality control of a single carbene molecule by tip-induced van der Waals interactions, *Nat. Commun.*, 2023, **14**, 4500.
14. H. Noei, H. Qiu, Y. Wang, E. Löffler, C. Wöll and M. Muhler, The identification of hydroxyl groups on ZnO nanoparticles by infrared spectroscopy, *Phys. Chem. Chem. Phys.*, 2008, **10**, 7092-7097.
15. D. Cappus, C. Xu, D. Ehrlich, B. Dillmann, C. A. Ventrice, K. Al Shamery, H. Kuhlenbeck and H. J. Freund, Hydroxyl groups on oxide surfaces: NiO(100), NiO(111) and Cr_2O_3(111), *Chem. Phys.*, 1993, **177**, 533-546.
16. M. Abu Haija, S. Guimond, A. Uhl, H. Kuhlenbeck and H. J. Freund, Adsorption of water on thin V_2O_3(0001) films, *Surf. Sci.*, 2006, **600**, 1040-1047.
17. J. Q. Zhong, Z. K. Han, K. Werner, X. Y. Li, Y. Gao, S. Shaikhutdinov and H. J. Freund, Water-Assisted Homolytic Dissociation of Propyne on a Reduced Ceria Surface, *Angew. Chem. Int. Ed.*, 2020, **59**, 6150-6154.
18. S. Kaya, J. Weissenrieder, D. Stacchiola, S. Shaikhutdinov and H. J. Freund, Formation of an ordered ice layer on a thin silica film, *J. Phys. Chem. C*, 2006, **111**, 759-764.
19. P. Dementyev, K. H. Dostert, F. Ivars-Barcelo, C. P. O'Brien, F. Mirabella, S. Schauermann, X. Li, J. Paier, J. Sauer and H. J. Freund, Water interaction with iron oxides, *Angew. Chem. Int. Ed.*, 2015, **54**, 13942-13946.
20. C. Lemire, R. Meyer, V. E. Henrich, S. Shaikhutdinov and H. J. Freund, The surface structure of Fe_3O_4(111) films as studied by CO adsorption, *Surf. Sci.*, 2004, **572**, 103-114.
21. P. Costa, M. Fernandez-Oliva, E. Sanchez-Garcia and W. Sander, The highly reactive benzhydryl cation isolated and stabilized in water ice, *J. Am. Chem. Soc.*, 2014, **136**, 15625-15630.
22. A. A. Kananenka and J. L. Skinner, Fermi resonance in OH-stretch vibrational spectroscopy of liquid water and the water hexamer, *J. Chem. Phys.*, 2018, **148**, 244107.
23. J. Carrasco, A. Hodgson and A. Michaelides, A molecular perspective of water at metal interfaces, *Nat. Mater.*, 2012, **11**, 667-674.

24. T. Schiros, S. Haq, H. Ogasawara, O. Takahashi, H. Öström, K. Andersson, L. G. M. Pettersson, A. Hodgson and A. Nilsson, Structure of water adsorbed on the open Cu(110) surface: H-up, H-down, or both?, *Chem. Phys. Lett.*, 2006, **429**, 415-419.
25. C. Clay, S. Haq and A. Hodgson, Intact and dissociative adsorption of water on Ru(0001), *Chem. Phys. Lett.*, 2004, **388**, 89-93.
26. K. Morgenstern, H. Gawronski, M. Mehlhorn and K.-H. Rieder, Local investigation of electron-induced processes in water-metal systems, *J. Mod. Opt.*, 2004, **51**, 2813-2819.
27. H. Gawronski, J. Carrasco, A. Michaelides and K. Morgenstern, Manipulation and control of hydrogen bond dynamics in absorbed ice nanoclusters, *Phys. Rev. Lett.*, 2008, **101**, 136102.
28. G. Kichin, C. Weiss, C. Wagner, F. S. Tautz and R. Temirov, Single molecule and single atom sensors for atomic resolution imaging of chemically complex surfaces, *J. Am. Chem. Soc.*, 2011, **133**, 16847-16851.
